# *Listeria monocytogenes* 10403S Arginine Repressor ArgR Finely Tunes Arginine Metabolism Regulation under Acidic Conditions

**DOI:** 10.3389/fmicb.2017.00145

**Published:** 2017-01-31

**Authors:** Changyong Cheng, Zhimei Dong, Xiao Han, Jing Sun, Hang Wang, Li Jiang, Yongchun Yang, Tiantian Ma, Zhongwei Chen, Jing Yu, Weihuan Fang, Houhui Song

**Affiliations:** ^1^College of Animal Science and Technology, China-Australia Joint-Laboratory for Animal Health Big Data Analytics, Zhejiang Provincial Engineering Laboratory for Animal Health Inspection & Internet Technology, Zhejiang A&F UniversityLin’an, China; ^2^Zhejiang Provincial Key Laboratory of Preventive Veterinary Medicine, Institute of Preventive Veterinary Medicine, Zhejiang UniversityHangzhou, China

**Keywords:** *Listeria monocytogenes*, arginine repressor, ArgR, regulation, acid tolerance

## Abstract

*Listeria monocytogenes* is able to colonize human and animal intestinal tracts and to subsequently cross the intestinal barrier, causing systemic infection. For successful establishment of infection, *L. monocytogenes* must survive the low pH environment of the stomach. *L. monocytogenes* encodes a functional ArgR, a transcriptional regulator belonging to the ArgR/AhrC arginine repressor family. We aimed at clarifying the specific functions of ArgR in arginine metabolism regulation, and more importantly, in acid tolerance of *L. monocytogenes*. We showed that ArgR in the presence of 10 mM arginine represses transcription and expression of the *argGH* and *argCJBDF* operons, indicating that *L. monocytogenes* ArgR plays the classical role of ArgR/AhrC family proteins in feedback inhibition of the arginine biosynthetic pathway. Notably, transcription and expression of *arcA* (encoding arginine deiminase) and *sigB* (encoding an alternative sigma factor B) were also markedly repressed by ArgR when bacteria were exposed to pH 5.5 in the absence of arginine. However, addition of arginine enabled ArgR to derepress the transcription and expression of these two genes. Electrophoretic mobility shift assays showed that ArgR binds to the putative ARG boxes in the promoter regions of *argC, argG, arcA*, and *sigB*. Reporter gene analysis with *gfp* under control of the *argG* promoter demonstrated that ArgR was able to activate the *argG* promoter. Unexpectedly, deletion of *argR* significantly increased bacterial survival in BHI medium adjusted to pH 3.5 with lactic acid. We conclude that this phenomenon is due to activation of *arcA* and *sigB*. Collectively, our results show that *L. monocytogenes* ArgR finely tunes arginine metabolism through negative transcriptional regulation of the arginine biosynthetic operons and of the catabolic *arcA* gene in an arginine-independent manner during lactic acid-induced acid stress. ArgR also appears to activate catabolism as well as *sigB* transcription by anti-repression in an arginine-dependent way.

## Introduction

*Listeria monocytogenes* is a foodborne bacterial pathogen capable of invasion and replication in phagocytic and non-phagocytic cells. This capacity allows it to cross protective epithelial barriers of the human body and cause severe infection with high mortality, especially in elderly populations, infants, immunocompromised individuals, and pregnant women (Cossart, 2011; Lebreton et al., 2015). The bacterium is resistant to acidic environments encountered during food processing, in acidic food, the stomach and phagosomes of macrophages (Cotter and Hill, 2003; Gray et al., 2006), and employs several mechanisms for pH homeostasis to survive or even proliferate in acidic conditions. *L. monocytogenes* utilizes the arginine deiminase (ADI) and agmatine deiminase (AgDI) systems to produce ammonia to neutralize intracellular protons by forming NH4^+^ to elevate its cytoplasmic pH (Ryan et al., 2009; Chen et al., 2011; Cheng et al., 2013a,b). The general stress responsive alternative sigma factor sigma B (SigB or σB), which was first identified in *Bacillus subtilis* (Boylan et al., 1993), plays a pivotal role in response to environmental stresses in *Listeria* (Ferreira et al., 2001; Xia et al., 2016).

Arginine catabolism *via* the ADI pathway is widely distributed in bacteria enabling them to survive under harsh acidic environments and to evade host defenses (Marquis et al., 1987; Gruening et al., 2006; Lucas et al., 2007; Fulde et al., 2011; Xiong et al., 2015). The ADI pathway consists of three enzymes: ADI, ornithine carbamoyl-transferase and carbamate kinase, which are encoded by *arcA, arcB* and *arcC*, respectively. The actions of these three proteins convert arginine to ornithine, ammonia and carbon dioxide (Xiong et al., 2015). In many bacteria, the ADI pathway is regulated by an arginine repressor, ArgR, a hexameric protein that belongs to the ArgR/AhrC family of transcriptional regulators involved in regulation of arginine biosynthetic metabolism in a feedback manner (Fulde et al., 2011; Choi et al., 2012; Xiong et al., 2015). Classical biosynthesis of arginine from glutamate is composed of eight enzymatic steps (**Supplementary Figure S1A**). Five steps involving *N*-acetylated intermediates contribute to formation of ornithine, and three additional steps are required to convert ornithine into arginine *via* several biosynthetic enzymes encoded by *argABCDEFGH* (Cunin et al., 1986). In the recycling pathway as In bacilli and most other prokaryotes, the acetyl group of *N*-acetylornithine is effectively transferred to glutamate by an acetyltransferase (ArgJ), making *N*-acetylglutamate synthase (ArgA), and *N*-acetylornithinase (ArgE) of the linear pathway redundant (Lu, 2006). This situation is also found in *L. monocytogenes* (**Supplementary Figure S1B**). Generally, ArgR proteins regulate their target genes by binding to the operator sites (called ARG box), leading to repression of arginine biosynthetic genes and activation of catabolic genes in the presence of arginine. ArgR proteins also regulate various genes involved in arginine transport (Maghnouj et al., 1998; Caldara et al., 2006). ArgR-mediated regulation network has been shown to respond to various environmental stimuli, such as changes in concentration of arginine and other metabolites and fluctuations in pH, temperature, and oxygen tension (Dong et al., 2004; Gruening et al., 2006; Xiong et al., 2015).

Homologs of the ADI and the arginine biosynthesis pathway genes have been found in the sequenced genome of *L. monocytogenes* strain EGD-e by *in silico* analysis (Glaser et al., 2001). The ADI encoded by *arcA*, is a critical enzyme in the ADI system that triggers the first reaction. The molecular characteristics of the ADI system and its contributions to acid tolerance of *L. monocytogenes in vitro* have been studied (Ryan et al., 2009; Cheng et al., 2013b), but the underlying regulatory mechanisms have not been determined. Moreover, SigB is an important component that links survival to environmental stress and virulence in *L. monocytogenes* and is involved in the regulation of more than 150 genes (Palmer et al., 2011; Xia et al., 2016). Nevertheless, little is known about the regulation of transcription and expression of SigB in *L. monocytogenes*. Here, we report that *L. monocytogenes* ArgR is a negative regulator of the expression, not only of arginine biosynthesis genes (*argCJBD* and *argGH*) but also of ArcA, essential for arginine catabolism, and of SigB. Such regulation might occur by direct interaction with its ARG boxes in the promoter regions. Most notably, we determined that ArgR plays a unique role in acidic tolerance of *L. monocytogenes* by exerting a regulatory role on *arcA* and *sigB*.

## Materials and Methods

### Bacterial Strains, Plasmids, and Culture Conditions

*Listeria monocytogenes* 10403S was used as the wild-type strain. *Escherichia coli* DH5α was employed for cloning experiments and as the host strain for plasmids pET30a(+; Merck, Darmstadt, Germany), pERL3 and pKSV7. *E. coli* Rosetta (DE3) was used for prokaryotic protein expression. *L. monocytogenes* was cultured in brain heart infusion (BHI) medium (Oxoid, Hampshire, England). DH5α and Rosetta (DE3) cells were grown at 37°C in Luria-Bertani broth (LB; Oxoid). Stock solutions of ampicillin (50 mg/ml), erythromycin (50 mg/ml), kanamycin (50 mg/ml), or chloramphenicol (50 mg/ml) were added to media when necessary. All chemicals were obtained from Sangon Biotech (Shanghai, China), Merck or Sigma-Aldrich (St. Louis, MO, USA) and were of the highest available purity.

### Bioinformatics Analysis

Alignment of nucleotide and deduced amino acid sequences was performed with MUSCLE by using Geneious software (Edgar, 2004). The amino acid sequences of ArgR of *L. monocytogenes* 10403S strain and its homologs in other microbial species were obtained from the National Centre for Biotechnology Information database (NCBI). The known crystal structure of *B. subtilis* ArgR (PDB: 1F9N) was acquired from the Protein Data Bank (PDB). A putative model of *L. monocytogenes* ArgR was constructed using SWISS-MODEL Workspace (Arnold et al., 2006; Bordoli et al., 2009; Bordoli and Schwede, 2012). Promoters of genes of interest from the *L. monocytogenes* 10403S genome sequence were identified using the BPROM modules of the Softberry website^1^. This program gives output scores from -1 to ∼25 to estimate the likelihood that a predicted promoter is functional and a higher score indicates that the prediction is more likely to be correct. ArgR binding sites composed of two palindrome sequences, known as ARG boxes, have been identified previously in several bacteria species (Larsen et al., 2005; Kloosterman and Kuipers, 2011; Perez-Redondo et al., 2012). Promoter/operator elements containing ARG box motifs were identified by searching the *L. monocytogenes* genome with a position weight matrix derived from known *E. coli* ArgR recognition elements (Chen et al., 1997), using the Virtual Footprint software program^2^ (Munch et al., 2005).

### Construction of Gene Deletion Mutant

The temperature-sensitive pKSV7 shuttle vector was used for generating mutations in *L. monocytogenes* 10403S. A homologous recombination strategy with the splicing by overlap extension (SOE) PCR procedure was used for in-frame deletion to construct gene deletion mutants (Monk et al., 2008). DNA fragments containing homologous arms upstream and downstream of the gene of interest were obtained via amplification of 10403S genomic DNA using the primer pairs listed in Supplementary Table S1. The obtained fragment was then cloned into pKSV7 and transformed into DH5α. After confirmation by sequencing, the recombinant vector containing the target gene deletion cassette was electroporated into the competent *L. monocytogenes* cells. Transformants were selected on BHI agar plates containing chloramphenicol (10 μg/ml). A single transformant was serially passaged at a non-permissive temperature (41°C) in BHI medium containing chloramphenicol to promote chromosomal integration. A single colony with chromosomal integration was successively passaged in BHI medium without chloramphenicol at a permissive temperature (30°C) to enable plasmid excision and curing (Camilli et al., 1993). Recombinants were identified as chloramphenicol-sensitive colonies, and mutagenesis was further confirmed by PCR and DNA sequencing. The single mutant strain was used in a second round of mutagenesis to construct double deletion mutants.

### Complementation of *argR* Deletion Mutant

To complement the *L. monocytogenes*ΔArgR strain, the complete *argR* open reading frame (ORF) along with its promoter was amplified from genomic DNA using primer pairs listed in Supplementary Table S1. After digestion with appropriate enzymes, the PCR product was cloned into pERL3, a plasmid capable of replication in Gram positive bacteria. The resulting plasmid was then electroporated into the *L. monocytogenes* ΔArgR strain. Plasmid-containing cells were selected on BHI agar plates containing erythromycin (10 μg/ml). The complemented strain was designated as CΔArgR.

### Expression and Purification of Recombinant Proteins

The recombinant proteins used in this study were expressed as fusion proteins to the N-terminal His-tag using pET30a(+) as the expression vector. Rosetta (DE3) was used as the expression host. The full-length ORF of the gene of interest from the 10403S genome was amplified with the primer pair listed in Supplementary Table S1 and inserted into the pET30a(+) vector, and finally transformed into Rosetta competent cells. *E. coli* cells harboring recombinant plasmids were grown in 250 mL LB supplemented with 50 μg/mL kanamycin at 37°C until cultures reached 1.2–1.4 at OD_600__nm_. Isopropyl β-D-1-thiogalactopyranoside (IPTG) was added to a final concentration of 0.4 mM to induce expression of interest proteins for an additional 3–4 h at 30°C in the form of soluble protein. His-tagged fusion proteins were purified using nickel-chelated affinity column chromatography (Weishi-Bohui Chromtotech Co., Beijing, China). Specifically, IPTG-induced cell pellets were collected, re-suspended in 50 mM PBS (pH 7.4) and disrupted by sonication. After centrifugation at 12,000 g for 30 min, the soluble protein samples were collected and loaded onto a 1 ml pre-packed nickel-chelated agarose gel column according to the manufacturer’s instructions. Finally, expression and purification of recombinant proteins were analyzed via 10% SDS-PAGE followed by Coomassie brilliant blue staining and protein concentration was quantified with the Bradford method.

### Preparation of Polyclonal Antibodies against Recombinant Proteins

Rabbits were initially immunized via subcutaneous injection of 500 μg protein with an equal volume of Freund’s complete adjuvant (Sigma). After 2 weeks, rabbits were given subcutaneous booster injections of 250 μg protein each in incomplete Freund’s adjuvant (Sigma) three times at 10-day intervals. Rabbits were bled ∼10 days after the last injection.

### Producing a Truncated ArgR by Site-Directed Mutagenesis

To identify the predicted active sites of ArgR, a double mutant (S42AR43A) was generated using the original vector template, pET30a-ArgR, and the QuikChange Site-Directed Mutagenesis kit (Agilent, Santa Clara, CA, USA) with the primer pairs described in Supplementary Table S1. Template DNA was removed via digestion with *Dpn*I (TOYOBO, Osaka, Japan) for 2 h at 37°C. The mutant construct was sequenced to ensure that only the desired single mutations had been incorporated correctly. The corresponding mutant protein was designated ArgR_S42AR43A_, and expressed and purified as described above.

### Crosslinking Analysis

The purified N-terminal 6-histidine-tagged ArgR proteins were crosslinked with various amounts of glutaraldehyde (Sigma) in 50 mM HEPES (pH 8.0), containing 150 mM KCl and 1 mM L-arginine. The reaction mixture was incubated with or without 1% β-mercaptoethanol at room temperature for 2 h and the cross-linked ArgR complexes were analyzed by 10% SDS-PAGE, and stained with Coomassie Brilliant Blue.

### Electrophoretic Mobility Shift Assay (EMSA)

DNA binding of ArgR and its mutant ArgR_S42AR43A_ was investigated *in vitro* by using electrophoretic mobility shift assay (EMSA). The DNA fragment of the promoter region of *argC, argG, arcA, or sigB* containing the putative ARG box was generated by PCR with the specific primer pairs (Supplementary Table S1). The DNA fragments were purified with a PCR Purification Kit (Sangon). Then 200 ng DNA was incubated with varying concentrations of purified recombinant ArgR or ArgR_S42AR43A_ in binding buffer (50 mM Tris-HCl, pH 8.0, 250 mM NaCl, 5.0 mM MgCl_2_, 2.5 mM DTT, 2.5 mM EDTA, and 20% glycerol) for 30 min at room temperature. Protein-DNA complexes were separated electrophoretically on a native 5% polyacrylamide gel at 80 V with 0.5 x Tris-acetate-EDTA (TAE) buffer and visualized using ethidium bromide staining.

### Construction of P*_argG_* Fusing *gfp* Reporter Strains and Promoter Studies

For transcriptional fusion of the *argG* promoter (P*_argG_*) to the GFP reporter protein, the fragment containing the promoter-operator region of the *argGH* operon was amplified with the primer pair listed in Supplementary Table S1 using genomic DNA from *L. monocytogenes* 10403S as template. In parallel, the promoterless *gfp* allele *gfp*mut3^∗^ was amplified from the *Listeria* shuttle vector pAMGFP3 using primers listed in Supplementary Table S1. The two fragments were fused by using overlapping PCR. The resulting fragment containing the promoter-*gfp* fusion was cloned into vector pERL3 to generate the reporter plasmid which was then electroporated into the wild-type 10403S or the ΔArgR strain. Transformants were selected by plating onto erythromycin-containing BHI agar plates. For promoter studies, *L. monocytogenes* was grown to stationary phase (OD_600__nm_ = 1.2) in BHI broth at 37°C, and then exposed to acidic (pH 5.5) or neutral (pH 7.0) conditions for an additional 60 min. Bacteria in 1 mL of culture were harvested by centrifugation, the cell pellets were washed once with 10 mM PBS (pH 7.4) and resuspended in 1 mL of 10 mM PBS. One hundred microliters of the suspension was used for *gfp* measurements and fluorescence observation. For the former, relative fluorescence unites (RFU) were measured in a fluorescence reader (BioTek Synergy H1, Winooski, VT, USA) with excitation at 485 nm and emission at 535 nm. Relative fluorescence values were calculated by subtracting extinction from the PBS background. For the latter, fluorescence intensity was observed by using confocal laser scanning microscopy (FLV 1000; Olympus, Japan).

### Survival in Acidic Conditions

Cells from stationary phase cultures of *L. monocytogenes* 10403S, mutants (ΔArgR, ΔSigB, and ΔArgRΔSigB) and complemented strain CΔArgR were harvested, washed in PBS and re-suspended in BHI (adjusted to pH 3.5 with 3M lactic acid). After 30, 60, 90, 120,160, or 200 min of incubation at 37°C, the surviving cells were plated onto BHI agar after appropriate dilutions. The plates were incubated at 37°C for 24 h and survival rates are reported as the mean of three independent experiments, which were performed in duplicate.

### Real-Time Quantitative RT-PCR (qRT-PCR)

*Listeria monocytogenes* wild-type 10403S and its mutant strain ΔArgR were grown to the stationary phase (OD_600__nm_ = 1.2) in BHI broth at 37°C, and then exposed to acidic (pH 5.5) and neutral (pH 7.0) conditions, respectively, for additional 1 h. Total RNA was extracted using the Column Bacterial total RNA Purification Kit (Sangon), according to the manufacturer’s instructions, genomic DNA removed using DNase I (TaKara, Japan) and cDNA synthesized with reverse transcriptase (TOYOBO, Osaka, Japan). Real-time quantitative PCR was performed in a 20 μL reaction volume containing 200 ng cDNA, 10 μL SYBR quantitative PCR mix (TOYOBO), and 1 μL gene-specific primers (200 nM, Supplementary Table S1) to measure the transcriptional levels of *arcA, sigB, argC*, and *argG* using the Mx3000P PCR detection system (Agilent). The housekeeping gene, *gyrB*, was used as an internal control for normalization in each sample as previously described (Chen et al., 2011). Relative transcription levels were quantified using the 2^-ΔΔCT^ method and shown as relative fold changes (Livak and Schmittgen, 2001). Triplicate assays were performed for each gene.

### Preparation of Whole-Cell Lysates and Western Blot Analysis

Bacteria were grown in BHI broth to the stationary growth phase and lysates were prepared as described before (Ryan et al., 2009). Specifically, the stationary bacteria exposed to acidic (pH 5.5) and neutral (pH 7.0) conditions, respectively, for additional 1 h. Bacterial pellets were then re-suspended in 1 mL of extraction solution (2% Triton X-100, 1% SDS, 100 mM NaCl, 10 mM Tris-HCl, 1 mM EDTA, pH 8.0). One gram of glass beads (G8772, Sigma) was added and samples lysed using the homogenizer Precellys 24 (Bertin, Provence, France) at 6000 rpm for 30 s with intermittent cooling for 30 s (two cycles in total), followed by centrifugation at 12,000 g for 15 min. Supernatants were retained as cell-free extracts. Samples containing equal amounts of protein were subjected to 12% SDS-PAGE and the separated proteins were blotted onto 0.22 μm polyvinylidene difluoride (PVDF) membranes (Merck Millipore). Membranes were blocked for 1 h with 5% skimmed milk, and incubated for 1 h with polyclonal antisera against recombinant recombinant ArgR, ArgG, SigB, or ArcA in 0.5% skimmed milk. Next, membranes probed with anti-interest protein were developed using HRP-conjugated goat anti-rabbit IgG (Santa Cruz, California, CA, USA) as the secondary antibody. Chemiluminescence was detected via a bio-imaging system (UVP EC3 Imaging System, UVP Inc., Upland, CA, USA), and the densities of the interest protein bands were normalized to the GAPDH signal and quantified using Quantity One software (Bio-Rad).

### Statistical Analysis

All data comparisons were analyzed using the two-tailed Student *t-*test. Differences with *P-*values of 0.05 were considered statistically significant, and those with *P-*values of 0.01 were considered markedly statistically significant.

## Results

### *L. monocytogenes* ArgR Is Predicted as a Typical Arginine Repressor

**FIGURE 1:**
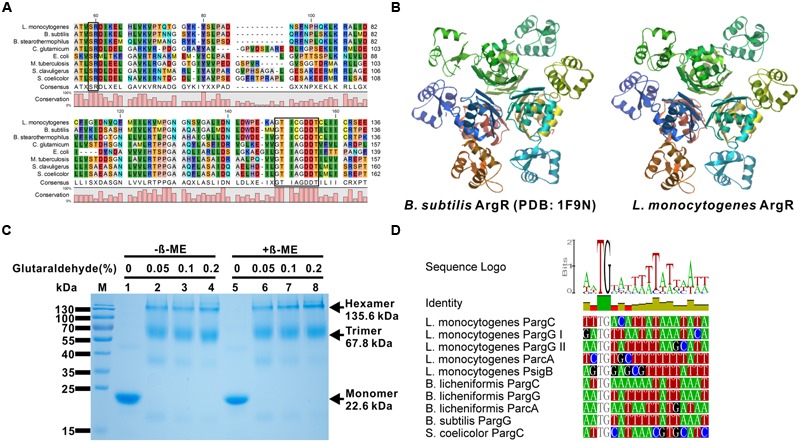
***Listeria monocytogenes* ArgR protein is a member of ArgR/AhrC family transcriptional regulators. (A)** Amino acid sequence alignment of *L. monocytogenes* ArgR against the members of the ArgR/AhrC family from *Bacillus subtilis, B. stearothermophilus, Corynebacterium glutamicum, Escherichia coli, Mycobacterium tuberculosis, Streptomyces clavuligerus*, and *S. coelicolor*. The two conserved motifs that are responsible for DNA and arginine binding are blackened. **(B)** Predicted tertiary fold of *L. monocytogenes* ArgR using the *B. subtilis* ArgR (PDB: 1F9N) as the template in the SWISS-MODEL Workspace. **(C)** SDS-PAGE analysis of glutaraldehyde crosslinking of *L. monocytogenes* ArgR for the identification of protein polymers. The monomeric, trimeric and hexameric proteins are indicated by arrows. **(D)** Promoter/operator elements containing binding sites of ArgR (ARG box) were identified by searching the *L. monocytogenes* genome with a position weight matrix derived from known ArgR recognition elements using the Virtual Footprint software program (as described in detail in the Materials and Methods). The identified potential consensus binding sites of ArgR in the promoter region from *L. monocytogenes* gene *argC, argG, arcA*, and *sigB* were further aligned with those from *B. subtilis, B. licheniformis*, and *S. coelicolor*.

The best characterized ArgR homolog from Gram-positive bacteria is AhrC from *B. subtilis*, as its crystal structure was determined in 2002 (PDB ID: 1F9N; Garnett et al., 2007). To better characterize *L. monocytogenes* ArgR, the amino acid sequence of this protein was aligned with those from seven other bacterial species. The alignment showed sequence identities ranging from 22 to 65%, the highest percentage was exhibited between ArgR from *L. monocytogenes* and AhrC from *B. subtilis* (**Figure 1A**). Based on the amino acid sequence analysis, the *L. monocytogenes* ArgR monomer appears to possess two highly conserved motifs, the “SR” motif for DNA binding (residues 42–43) in the N-terminal region, and the “GTICGDDT” motif for arginine binding (residues 120–127) and oligomerization (residues 125–126) located in the C-terminal region (**Figure 1A**). Furthermore, we modeled *L. monocytogenes* ArgR in the SWISS-MODEL Workspace using the crystal structure of *B. subtili*s AhrC as the template. The predicted structure *L. monocytogenes* ArgR is of high similarity to that of AhrC. Specifically, the monomer ArgR also associates via its C-terminal (core) domain to form a hexamer, probably as a result of the face-to-face association of a pair of trimers (Dennis et al., 2002). The six C-terminal domains strictly follow the 32 non-crystallographic symmetry (NCS) and domains from each trimer locate above another one, giving the hexameric core a stacked configuration when viewed along the threefold axis. The DNA-binding domains (DBD) adopt slightly different positions around the periphery of the core and deviate from strict NCS (**Figure 1B**). The recombinant ArgR protein was expressed in *E. coli*, purified and subjected to crosslinking analysis using glutaraldehyde. The his-tagged ArgR protein had a molecular weight of about 23 kDa, and was able to form higher order multimeric complexes (mainly formed as the trimers and hexamers) in the presence of 0.05% glutaraldehyde (**Figure 1C**). These results suggest that ArgR of *L. monocytogenes* is a typical arginine repressor which might contribute to transcriptional regulation of arginine metabolism.

### ARG Boxes are Present in the Promoter Regions of *argCJBD, argGH, sigB*, and *arcA*

We further analyzed the sequences of the promoter regions of *argCJBD, argGH, sigB*, and *arcA* genes for possible ArgR binding sites using a virtual footprint promoter analysis program (see Materials and Methods; Munch et al., 2005). The ARG box consensus was described as TNTGAATWWWWATTCANW in *E. coli* (Maas, 1994), CATGAATAAAAATKCAAK in *B. subtilis* (Miller et al., 1997), and AWTGCATRWWYATGCAWT in *Streptomyces* (Rodriguez-Garcia et al., 1997; where W = A or T, K = G or T, R = A or G, Y = T or C, N = any base). Five putative ARG boxes were identified in each putative promoter region upstream of these five genes from *L. monocytogenes* on the basis of similarity with the *B. subtilis* (Makarova et al., 2001) and *B. licheniformis* (Maghnouj et al., 1998) and there were 1–3 bp mismatch with respect to the consensus sequence (**Figure 1D**).

### ArgR Binds *In vitro* to the ARG Boxes of *argCJBD, argGH, sigB*, and *arcA* Promoters

**FIGURE 2:**
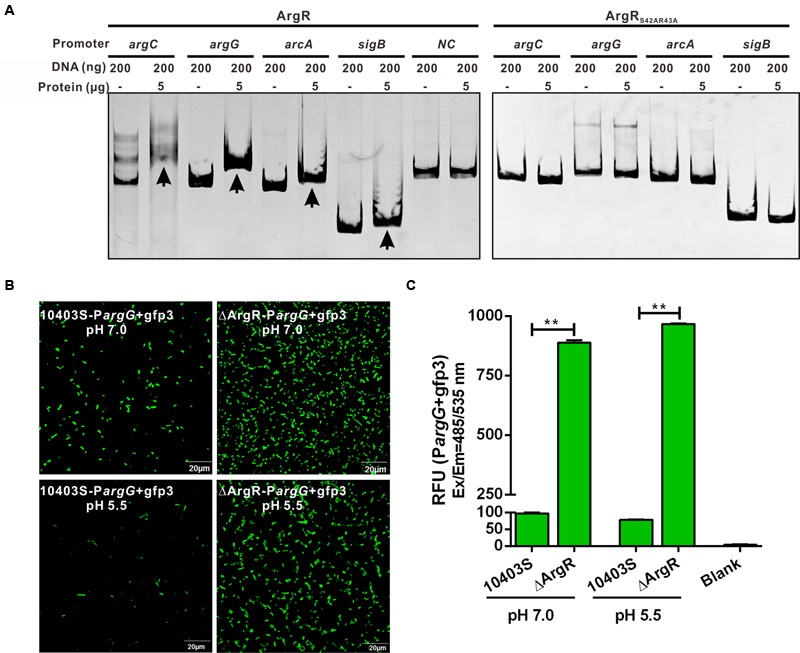
**(A)** ArgR binds *in vitro* to the operating sites of *argC, argG, arcA* and *sigB*, and activates the *argG* operon promoter. Gel mobility shift assay (EMSA) experiments were employed to examine binding of the recombinant ArgR from *L. monocytogenes* (ArgR) and its amino acid mutant protein (ArgR_S42AR43A_) to the *argC, argG, arcA*, and *sigB* promoter region DNA. The promoter fragments were obtained by PCR with primers specified in Supplementary Table S1, and incubated with recombinant proteins for 30 min at room temperature. Gel retardation by DNA–protein complexes was monitored after ethidium bromide staining. The housekeeping gene *gyrB* was used as a negative control (NC) for the EMSAs. Arrows indicate DNA-protein complexes. **(B,C)** ArgR activates the *argG* operon promoter. The fluorescence intensity was observed by confocal laser scanning microscopy of overnight grown *L. monocytogenes* wild-type and ArgR mutant strains carrying a *gfp3* reporter fused with the promoter of *argG*, and then stress-treated for an additional 1 h under pH 7.0 and 5.5 conditions **(B)**. Bars represent the relative fluorescence units (RFU) after subtracting the absolute values for the PBS control **(C)**. Data shown represents the Mean ± SD of three independent experiments, each performed in duplicate. ^∗∗^*P* < 0.01 for comparisons between the wild-type and mutant strains.

To confirm that ArgR directly binds to the respective ARG boxes identified above, the EMSA was performed. The promoter regions containing the putative ARG boxes were generated and incubated with recombinant ArgR, and the protein-DNA complexes were assayed by native gel electrophoresis. **Figure 2A** shows that recombinant ArgR was able to bind to DNA oligonucleotides of each promoter of *argCJBD, argGH, sigB*, or *arcA*. More significantly, ArgR shows stronger binding capacity to *argCJBD* and *argGH* promoters than to those of *sigB* and *arcA* under the experimental conditions we studied. These results indicate that *L. monocytogenes* ArgR contributes to overall regulation of the *argCJBD, argGH, sigB*, and *arcA* promoter activities although the degree of regulation could be different. More importantly, we found that ArgR protein completely lost the binding ability to ARG boxes when the two residues Ser42 and Arg43 (SR motif) were mutated to alanine (**Figure 2A**), strongly suggesting that these two sites are critical amino acids for ArgR-DNA binding.

### ArgR Activates the *argG* Operon Promoter

To further analyze the regulatory function of ArgR in expression of its target genes, we cloned a DNA fragment covering the promoter-operator region of the *argGH* operon (as the representative gene cluster involved in arginine anabolism) into the *gfp* reporter vector which was transformed into ΔArgR mutant and its parent strain. Data show that GFP expression was significantly elevated in the ΔArgR mutant under neutral and acidic conditions while GFP was barely detectable in the wild-type strain (**Figures 2B,C**). Thus, ArgR is shown to repress the arginine biosynthetic pathway by interacting with the promoter of the *argGH* operon.

### ArgR Regulates Transcription and Expression of *argC, argG, arcA*, and *sigB*

**FIGURE 3:**
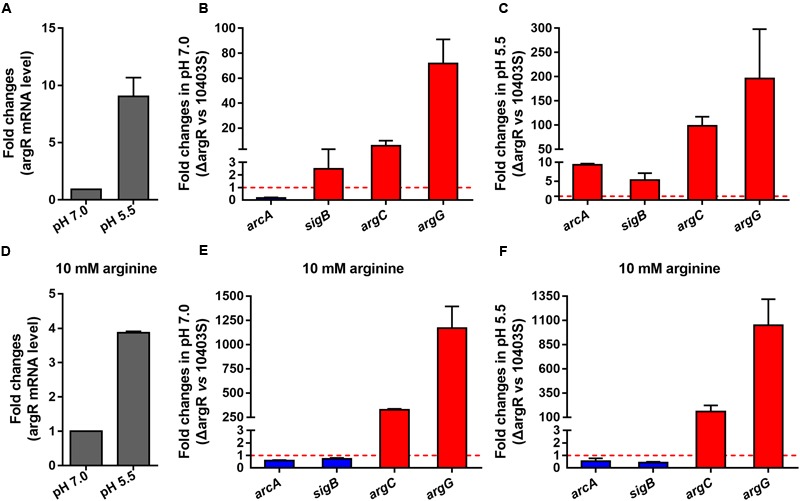
**ArgR regulates the transcription of *argC, argG, arcA*, and *sigB* using arginine as a cofactor.** Relative quantification of *argR, arcA, sigB, argC*, and *argG* mRNA and protein expression levels in *L. monocytogenes* wild-type and ΔArgR mutant strains under different pH conditions (7.0 and 5.5) in the presence **(A–C)** or absence **(D–F)** of exogenous arginine (10 mM). Values are expressed as Mean ± SD. The dotted lines indicate the onefold change in transcription of the interest genes.

Since the putative ARG boxes are present in the promoter regions of *argC, argG, arcA*, and *sigB*, transcription of these genes might be regulated by ArgR. To find out if such regulation occurs, real-time quantitative PCR was performed using total RNA isolated from the wild-type strain *L. monocytogenes* 10403S and the *argR* deletion mutant ΔArgR in the presence (10 mM) or absence of arginine. We found that expression of *argR* was significantly induced in response to acidic pH at 5.5 regardless of arginine supplementation (**Figures 3A,D**). The transcriptional levels of two representative genes (*argC and argG)* involved in arginine anabolism were significantly increased in the ΔArgR mutant under neutral or acidic conditions (**Figures 3B,C**), and such effects were augmented by addition of exogenous arginine (**Figures 3E,F**). These findings indicate that *L. monocytogenes* ArgR plays a classical role of ArgR/AhrC family in feedback inhibition of the arginine biosynthetic pathway using arginine as a corepressor. Transcription of *arcA* was downregulated in the ΔArgR mutant under neutral pH (**Figure 3B**), which is consistent with findings from the previous study by Ryan et al. (2009), whereas *sigB* was slightly upregulated (**Figure 3B**). However, these two genes were markedly repressed by ArgR when bacterial cells were exposed to acidic pH in the absence of arginine (**Figure 3C**), but addition of arginine weakened the effect of ArgR on transcription of *arcA* and *sigB* regardless of pH conditions (**Figures 3E,F**). Therefore, *L. monocytogenes* ArgR appears to be a functional transcriptional regulator that modulates the expression of the *arc* operon positively and negatively under neutral and acidic pH conditions, respectively, by employing arginine as a cofactor.

**FIGURE 4:**
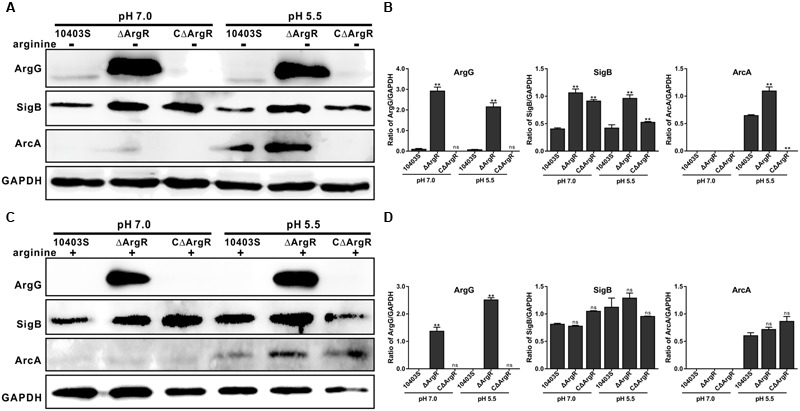
**ArgR regulates the expression of ArgG, SigB, and ArcA using arginine as a cofactor.** Total bacterial cell-free protein was isolated 2 h after stress under different pH conditions (7.0 and 5.5) in the absence **(A)** or presence **(C)** of exogenous arginine (10 mM), and the protein expression levels of ArgG, SigB, and ArcA were then determined by Western blotting. GAPDH was used as an internal control. The results are indicated as of the gray scale ratio of each interest protein to GADPH **(B,D)**. Data shown represents the Mean ± SD of two independent experiments. ^∗∗^*P* < 0.01; ns means no significance.

Immuno-blotting was used to determine the relevance of ArgR to the expression of arginine metabolism operon proteins ArgG and ArcA as well as SigB under neutral and acidic pH (5.5) conditions. Expression of ArgG in ΔArgR strain was significantly higher under neutral and acidic environments than that of the wild-type strain. (**Figures 4A,B**). When exogenous arginine was added, expression of ArgG was not detected in the wild-type strain, but expression strongly increased when ArgR was absent (**Figures 4C,D**), further indicating that arginine cooperates with ArgR to repress the arginine biosynthetic pathway in *Listeria*. In addition, ArgR can regulate the expression of ArcA and SigB in an arginine-dependent and independent manner (**Figure 4**), which is consistent with the results from transcriptional analysis mentioned above.

### Deletion of ArgR Enhances Survival of *L. monocytogenes* at Lethal Acidic pH

**FIGURE 5:**
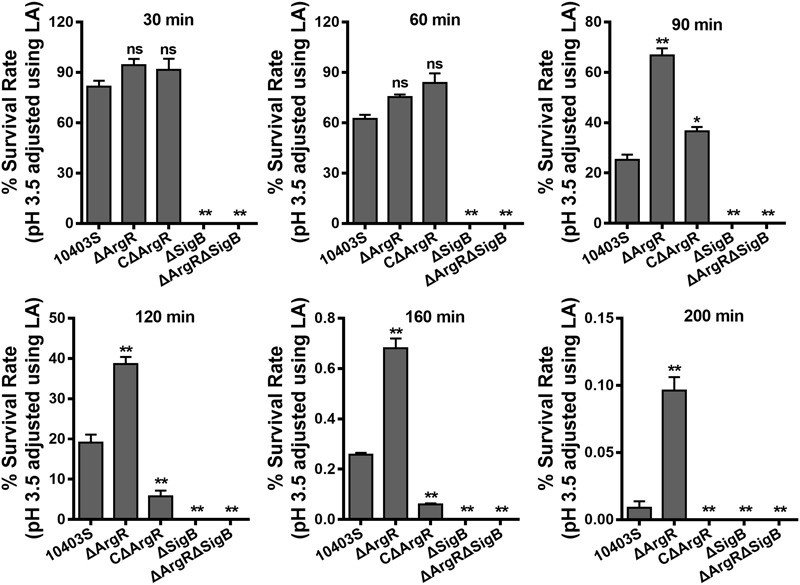
**Deletion of ArgR enhances survival of *L. monocytogenes* in the lethal acidic conditions.** Overnight-grown *L. monocytogenes* wild-type and mutant strains were harvested, washed and then incubated in BHI broth (pre-adjusted to pH 3.5 using 3 M lactic acid, LA) at 37°C. Survivors were enumerated at regular intervals by plating serial dilutions on BHI plate. Data are expressed as Mean ± SD of recovery rate for each strain. ^∗^*P* < 0.05; ^∗∗^*P* < 0.01; ns means no significance.

In order to investigate the contribution of ArgR to the survival of the bacterium at lethal pH values, acid tolerance experiments were carried out on the mutants in complex medium adjusted to a lethal pH of 3.5 using 3 M lactic acid. Data show that deletion of *argR* exhibited no significant difference in the rate of survival relative to the parent strain at the early time points (30 and 60 min; **Figure 5**). However, a notable increase in the number of surviving cells was observed for the ΔArgR from minutes 90 onward, as compared to those of the wild-type strain (**Figure 5**). Conversely, constitutive overexpression of ArgR compromised bacterial survival under the same pH conditions (**Figure 5**). It’s worth noting that these data are contradictory to findings by Ryan et al. (2009) who reported that *L. monocytogenes* ΔArgR had defect in acid resistance at both sublethal and lethal pH levels (Ryan et al., 2009). Nonetheless, based on our findings for ArgR involved in regulations on *arcA* and *sigB*, we speculated that increasing of the acidic survival in the absence of ArgR was most probably due to the activation *arcA* and *sigB*. In addition, consistent with our previous studies (Cheng et al., 2015), survival of *L. monocytogenes* was markedly compromised in the absence of *sigB* in the lethal acidic conditions (**Figure 5**).

## Discussion

The current study demonstrates that *L. monocytogenes* deploys ArgR to control arginine metabolism by negative regulation of arginine metabolism associated genes *via* binding to the putative ARG box operators as previously described (Fulde et al., 2011; Xiong et al., 2015). Structure modeling and oligomerization analysis indicate that *L. monocytogenes* ArgR has features similar to those of arginine repressors from other bacteria species, in particular with ArgR from *B. subtilis* (Dennis et al., 2002; Garnett et al., 2007). The N-terminal domain of ArgR is the DBD, whereas the C-terminal domain required for oligomerization and arginine binding (Sunnerhagen et al., 1997; Ni et al., 1999; Garnett et al., 2008). The ArgR protomers can form trimers and hexamers that are in equilibrium and their oligomerization state is manipulated by the presence of arginine corepressor that is bound in the space between ArgR trimers and link each pair of opposite trimers *via* their guanidinium groups, thereby providing additional stability as hexamer (Ni et al., 1999; Cherney et al., 2009).

As is the case in other bacteria species (Larsen et al., 2004; Nicoloff et al., 2004), we found that ArgR in *L. monocytogenes* also acts as a negative regulator of the arginine biosynthetic pathway by repression of *argCJBD* and *argGH* in the absence of ArgR, and such a regulatory effect was augmented under acidic conditions or in the presence of arginine. Generally, ArgR has been demonstrated to act as a positive regulator of *arcABC* operon expression in many bacteria species, which is essential for acid resistance (Griswold et al., 2004; Fulde et al., 2011; Xiong et al., 2015). ArcA and *sigB* were repressed by ArgR in the absence of extracellular arginine, while such effects were not seen when extracellular arginine was added. Notably, deletion of ArgR markedly enhanced the capacity of *L. monocytogenes* to survive in the lethal acid environments. However, Ryan et al. (2009) have previously noted that *L. monocytogenes* ΔArgR demonstrated a great defect in acid resistance at both sublethal and lethal pH levels. It’s worth noting that we used the same acidic conditions (media adjusted to pH 3.5 using 3 M lactic acid) and bacterial growth status as reported by Ryan et al. (2009); however, these authors used *L. monocytogenes* LO28, a serotype 1/2c strain (Ryan et al., 2009). We here speculate that the capacity of *L. monocytogenes* to survive in acidic environments was attributable to the activation of *arcA* and *sigB* in the absence of ArgR. It is well known that in a number of bacterial species, catabolism of arginine *via* the ADI pathway has been demonstrated to play a critical role in an enhanced capacity to survive under acidic extracellular conditions (Xiong et al., 2014; Xu et al., 2016), and *L. monocytogenes* is likely no exception (Ryan et al., 2009; Cheng et al., 2013b). Besides, the alternative factor, SigB has been widely studied as it plays a key role in *L. monocytogenes* survival under multiple environmental stress conditions, including elevated osmolarity, low pH and oxidative-stresses (Kim et al., 2004; Gahan and Hill, 2005).

This is the first study showing that a single ArgR regulator can have opposite regulatory effects on the ADI pathway in an arginine-independent and dependent manner under neutral and acidic conditions, respectively. However, the underlying molecular mechanisms are still unknown and warrant further study. In general, ArgR-type proteins act as a positive regulator of the ADI system and a negative regulator of the arginine biosynthetic pathway. However, there are two circumstances for unconventional ArgR regulatory mechanisms. One exists in bacteria that encode two ArgR homologs. For instance, the expression of arginine metabolism in *Lactococcus lactis* is controlled by the two homologous transcriptional regulators ArgR and AhrC. Specifically, ArgR binds to the promoter regions of both the arginine biosynthetic and catabolic operons in an arginine-independent manner. With both regulators present, addition of arginine leads to increased binding of ArgR-AhrC to the biosynthetic *argC* promoter but also to diminished binding to the catabolic *arcA* promoter (Larsen et al., 2005). The other circumstance is for the bacteria that contain one single ArgR homologous but two *arc* operons. Xiong et al. (2015) has demonstrated that arginine catabolism in *Laribacter hongkongensis* is finely regulated by manipulating the transcription of two *arc* operons. *L. hongkongensis* ArgR exhibited an opposite effect on transcription and expression of these two *arc* operons. In the presence of arginine, deletion of *argR* partially compromised the repressive effects that arginine had on *arcA1* expression; while it dramatically decreased the transcriptional levels of *arcA2* (Xiong et al., 2015).

**FIGURE 6:**
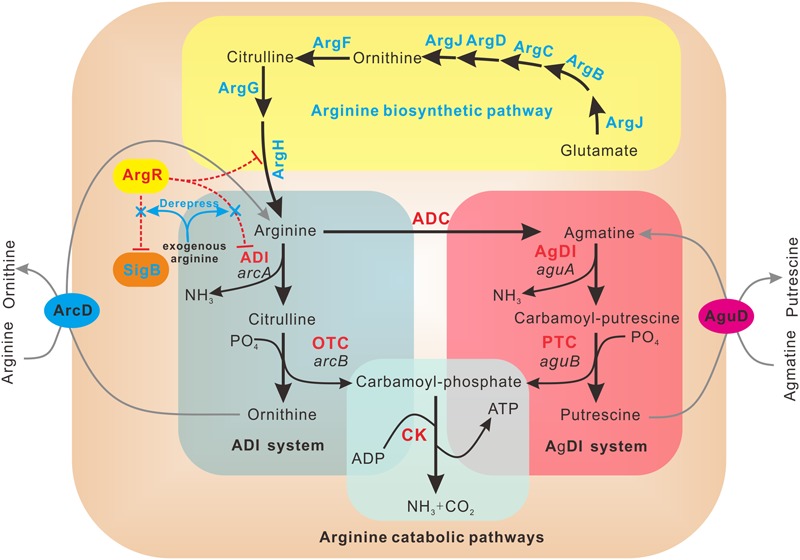
**Schematic representation of regulatory mechanism employed by ArgR in *L. monocytogenes*.** *L. monocytogenes* ArgR plays a classical role of ArgR/AhrC family in feedback inhibitory of arginine biosynthetic pathway (highlighted in yellow) using arginine as a cofactor. ArgR unexpectedly represses the transcription and expression of *arcA* and *sigB* in the absence of exogenous arginine, preventing arginine degradation *via* the ADI and AgDI pathways (highlighted in light cyan and light red, respectively). Addition of arginine leads to derepression of *arcA* and *sigB*, allowing utilization of arginine as a nitrogen and energy source *via* the arginine degradation pathway.

Since *L. monocytogenes* encodes a single ArgR homolog and one *arc* operon, we speculate that ArgR maintains its functions as a unique transcriptional regulator with dual regulatory effects on ADI pathway and SigB under different environmental stresses. Based on the results presented here, we propose a model depicting the mechanisms of ArgR in arginine-meditated transcriptional regulation in *L. monocytogenes* (**Figure 6**). In the absence of arginine, ArgR shows higher affinity for *arc* operon promoter, and relatively lower affinity for *arg* operons compared to that in the presence of arginine, consequently preventing arginine degradation *via* the ADI pathway and repressing arginine biosynthesis to a low extent. The addition of arginine shifts ArgR from the *arcA* promoter to the ARG box operators in the *arg* operons, which enhances repression of the arginine biosynthetic genes. Accordingly, the arginine catabolic *arc* operon is now derepressed, allowing catabolism of arginine as a nitrogen and energy source through the ADI arginine degradation pathway. Therefore, *L. monocytogenes* ArgR appears to have unusual roles in repression of arginine biosynthetic operon in an arginine-independent manner, and activation of catabolism by anti-repression in an arginine-dependent way.

## Author Contributions

CC, WF, and HS conceived the study. CC, JS, XH, ZD, HW, LJ, and TM carried out experiments. CC, YY, ZC, and JY analyzed data. CC, WF, and HS drafted the manuscript and all the authors contributed to preparing the final version of the manuscript. All authors read and approved the final manuscript.

## Conflict of Interest Statement

The authors declare that the research was conducted in the absence of any commercial or financial relationships that could be construed as a potential conflict of interest.

## Footnotes

1 http://www.softberry.com/

2 http://prodoric.tu-bs.de/

## Supplementary Material

FIGURE S1 **The arginine biosynthetic pathway and organization of the gene cluster in *L. monocytogenes*. (A)** The arginine biosynthetic pathway. Arginine is synthesized from glutamate in eight steps catalyzed by a series of enzymes encoded by *argABCDEFGH*. Five steps involving *N*-acetylated intermediates lead to ornithine, and three additional steps are required to convert ornithine into arginine. The synthesis of all enzymes is subject to repression by arginine, mediated by the repressor ArgR. **(B)** Genetic organization of the arginine biosynthesis pathway gene cluster in *L. monocytogenes* strain 10403S.
