# Supplementary figures and images for: Listeria monocytogenes 10403S Arginine Repressor ArgR Finely Tunes Arginine Metabolism Regulation under Acidic Conditions
Source: Front Microbiol. 2017 Jan 31;8:145. doi: 10.3389/fmicb.2017.00145 (PMC5291005; doi:10.3389/fmicb.2017.00145)

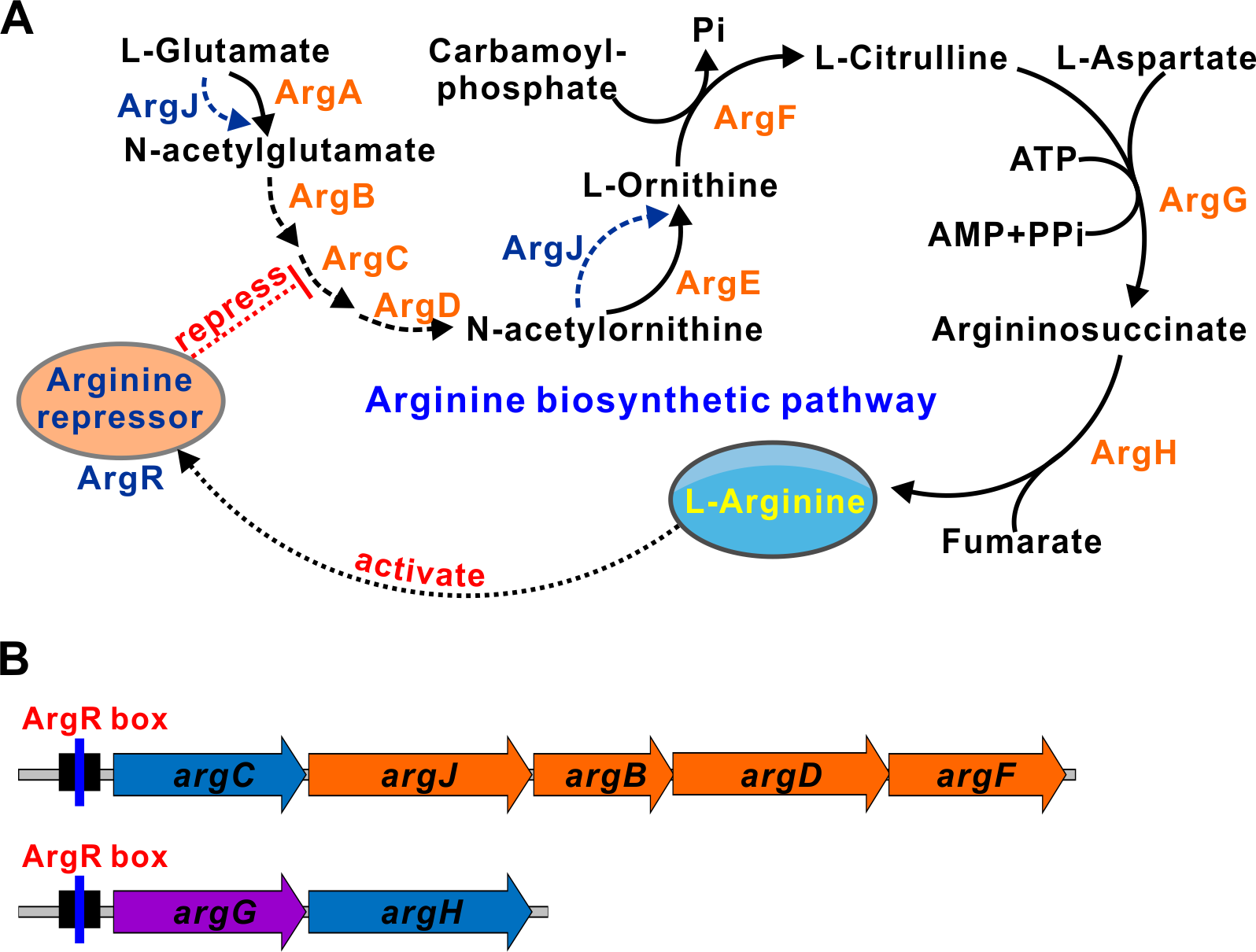

Supplement: FIGURE S1 — The arginine biosynthetic pathway and organization of the gene cluster in L. monocytogenes. (A) The arginine biosynthetic pathway. Arginine is synthesized from glutamate in eight steps catalyzed by a series of enzymes encoded by argABCDEFGH. Five steps involving N-acetylated intermediates lead to ornithine, and three additional steps are required to convert ornithine into arginine. The synthesis of all enzymes is subject to repression by arginine, mediated by the repressor ArgR. (B) Genetic organization of the arginine biosynthesis pathway gene cluster in L. monocytogenes strain 10403S. [file Image_1.TIF]
