# Supplementary material for: Listeria monocytogenes 10403S Arginine Repressor ArgR Finely Tunes Arginine Metabolism Regulation under Acidic Conditions
Source: Front Microbiol. 2017 Jan 31;8:145. doi: 10.3389/fmicb.2017.00145 (PMC5291005; doi:10.3389/fmicb.2017.00145)
Supplement: Supplementary file 2 [file Table_1.DOCX]

**Table S1.** PCR Primers used in this study. Nucleotides introduced to create restriction enzyme sites are underlined. All primers were synthesized by Sangon Biotech, Inc., Shanghai, China.

| **Primer name** | **Primer sequence (5’-3’)** | **Product (bp)** | **Description** |
| --- | --- | --- | --- |
| *argR*-a | TAGCATGCAACCACAATTTGAAGCAGGT | 476 | Used for construction of *argR* null mutant |
| *argR*-b | TCCTTCTTCACCTCACGATAATTTTCTA |  |  |
| *argR*-c | TGAGGTGAAGAAGGAAATTTGTTGGAGG | 519 |  |
| *argR*-d | TTGGTACCGTACTTGGTTAGTGCTGGCT |  |  |
| *sigB*-a | ATCTGCAGGAAATCACAGGATTGTCAG | 529 | Used for construction of *sigB* null mutant |
| *sigB*-b | AACTGCCTTGTTCATTCTCCTCCACCT |  |  |
| *sigB*-c | ATGAACAAGGCAGTTGAATCAAATAATTT | 561 |  |
| *sigB*-d | GCGAATTCTATCTAATATATTACGCTCGAT |  |  |
| CΔArgR-fwd | CGAGCTCTGTATACCGGAAAGCATTGATG | 656 | Used for complementation of the *argR* deletion |
| CΔArgR-rev | CGCGTCGACTTAAAGCATATCTATAAAACGATCTGT |  |  |
| ArgR-exp-fwd | CCGGAATTCATGAATAAAGGTCATCGTCATATTATTA | 462 | Used for recombinant ArgR expression in *E. coli* |
| ArgR--exp-rev | CCCAAGCTTTTAAAGCATATCTATAAAACGATCTGT |  |  |
| SigB-exp-fwd | CGCGGATCCATGCCAAAAGTATCTCAACCTGATAAAGAG | 792 | Used for recombinant SigB expression in *E. coli* |
| SigB-exp-rev | GCGGTCGACTTACTCCACTTCCTCATTCTGCAACG |  |  |
| ArgG-exp-fwd | CGCGGATCCATGGCGAAAGAAAAAATCGTATTAGCTTAC | 1227 | Used for recombinant ArgG expression in *E. coli* |
| ArgG-exp-rev | CCGCTCGAGTCACACCTCGGTTGTTATTATCACTTTTG |  |  |
| ArcA-exp-fwd | AGTGAATTCATGAAAATGGAACAA | 1251 | Used for recombinant ArcA expression in *E. coli* |
| ArcA-exp-rev | GCAGTCGACCTATTTTAGATTTTC |  |  |
| GAPDH-exp-fwd | CGCGGATCCATGACAGTTAAAGTTGGTAT | 1011 | Used for recombinant GAPDH expression in *E. coli* |
| GAPDH-exp-rev | CCGGAATTCTTATTTAGCGATTTTTGCAA |  |  |
| S42AR43A-fwd | ATGTTAAAGTCACCCAGGCAACTGTTGCCGCTGATATTAAAGAACTGC | 5859 | Used for site-directed mutagenesis of ArgR protein |
| S42AR43A-rev | GCAGTTCTTTAATATCAGCGGCAACAGTTGCCTGGGTGACTTTAACAT |  |  |
| P*_argG_* *gfp* reporter-a | CGCGAGCTCACGATTAATAATGGAAGTAGAATAATCCCAGC | 306 | Used for construction of P*_argG_* fusing *gfp* reporter |
| P*_argG_* *gfp* reporter-b | TTCTCCTTTACTCATTTTAAACATCCCCTATTCATAAATATACAAACATT |  |  |
| P*_argG_* *gfp* reporter-c | TAGGGGATGTTTAAAATGAGTAAAGGAGAAGAACTTTTCACTGGA | 741 |  |
| P*_argG_* *gfp* reporter-d | CGCGTCGACTTATTTGTATAGTTCATCCATGCCATGTGT |  |  |
| EMSA-P*_argC-_*fwd | AAATCGAGCATTACGAAAGCTTCACAG | 439 | Used for amplifying the promoter region DNA of *argC* |
| EMSA-P*_argC-_*rev | GGGCTAACTGCTAAATCTTTTAAATGTGG |  |  |
| EMSA-P*_argG-_*fwd | ATATCGCGAATCTACGTCGGATAATGGTG | 493 | Used for amplifying the promoter region DNA of *argG* |
| EMSA-P*_argG-_*rev | CCAACATCCAAGCAACATGCAATAACTTC |  |  |
| EMSA-P*_arcA-_*fwd | TGGAGCAAGCCGTATGAATTATCG | 429 | Used for amplifying the promoter region DNA of *arcA* |
| EMSA-P*_arcA-_*rev | TTCCTTCTGCATCATTTTTAAATACGGTA |  |  |
| EMSA-P*_sigB-_*fwd | GATCAATTAGTTAAAATGCAAAACTTTGA | 318 | Used for amplifying the promoter region DNA of *sigB* |
| EMSA-P*_sigB-_*rev | TTACCTTTGGCGCTGTATAAGCATC |  |  |
| EMSA-NC-fwd | AGGAAGAGTTAGCATATCAGAAACAGCT | 324 | Used for amplifying the negative control DNA |
| EMSA-NC-rev | CTGTTTCACCAAACTCTTTAATACTTGAA |  |  |
| *argR*-RT-fwd | CTAATGAGATTGACACGCAAGAAG | 80 | Used for transcriptional analysis of interest genes by RT-PCR |
| *argR*-RT-rev | GGAAACAGTTGCCTGGGTG |  |  |
| *argC*-RT-fwd | ACGATAATGCTCCATTTGTCCG | 82 |  |
| *argC*-RT-rev | TCGCAGTAATTTGAGGCTGTC |  |  |
| *argG*-RT-fwd | TTCAGCTTTAAGCCGTCCG | 93 |  |
| *argG*-RT-rev | TTACCAGTACAACCATGAGCGATA |  |  |
| *sigB*-RT-fwd | GGTGTCACGGAAGAAGAAG | 141 |  |
| *sigB*-RT-rev | TCCATCATCCGTACCACC |  |  |
| *arcA*-RT-fwd | CGATCTGTTAAGAAGTGCGGGTAT | 105 |  |
| *arcA*-RT-rev | TCTCTAACAAGCGGCATCGTCATAC |  |  |
| *gyrB*-RT-fwd | AGACGCTATTGATGCCGATGA | 91 |  |
| *gyrB*-RT-rev | GTATTGCGCGTTGTCTTCGA |  |  |
